# Prediction of Daily Blood Sampling Room Visits Based on ARIMA and SES Model

**DOI:** 10.1155/2020/1720134

**Published:** 2020-09-03

**Authors:** Xinli Zhang, Yu Yu, Fei Xiong, Le Luo

**Affiliations:** Department of Industrial Engineering and Engineering Management, Business School of Sichuan University, Chengdu 610065, China

## Abstract

This paper is aimed at establishing a combined prediction model to predict the demand for medical care in terms of daily visits in an outpatient blood sampling room, which provides a basis for rational arrangement of human resources and planning. On the basis of analyzing the comprehensive characteristics of the randomness, periodicity, trend, and day-of-the-week effects of the daily number of blood collections in the hospital, we firstly established an autoregressive integrated moving average model (ARIMA) model to capture the periodicity, volatility, and trend, and secondly, we constructed a simple exponential smoothing (SES) model considering the day-of-the-week effect. Finally, a combined prediction model of the residual correction is established based on the prediction results of the two models. The models are applied to data from 60 weeks of daily visits in the outpatient blood sampling room of a large hospital in Chengdu, for forecasting the daily number of blood collections about 1 week ahead. The result shows that the MAPE of the combined model is the smallest overall, of which the improvement during the weekend is obvious, indicating that the prediction error of extreme value is significantly reduced. The ARIMA model can extract the seasonal and nonseasonal components of the time series, and the SES model can capture the overall trend and the influence of regular changes in the time series, while the combined prediction model, taking into account the comprehensive characteristics of the time series data, has better fitting prediction accuracy than a single model. The new model can well realize the short-to-medium-term prediction of the daily number of blood collections one week in advance.

## 1. Introduction

In the Chinese medical context, the outpatient blood sampling room is the key node for clinical cases from diagnosis to the next step of treatment [1]. Often there are a series of challenges, such as too many blood-drawn patients and concentrated blood draw time, as well as a long waiting time, which not only reduce the patient's medical experience and satisfaction with the hospital, but also lower the work efficiency and job satisfaction of the nursing staff [2]. In recent years, with an increasing demand on the quality of medical services, improving the ability to predict the demand for outpatient blood collection medical services and forecasting the level of demand (patient flow) in advance (days, weeks) have a practical significance, especially for the allocation of medical resources and the satisfaction of high-quality needs.

As a hospital management decision support system, the outpatient service demand prediction system improves the overall efficiency of the outpatient department, thereby improving work efficiency and patient satisfaction [3]. In the actual operation of hospitals, many medical institutions do not perform demand forecasting, and those that conduct demand forecasting tend to use very basic methods with low accuracy. Therefore, scholars have paid more attention to the establishment of outpatient demand forecasting models. Existing research has focused on outpatient flow [4, 5] and other related departments like internal medicine [6, 7], cancer [8], anxiety disorder [3], diarrhea [9], and so on; however, the prediction of outpatient blood sampling room visits was ignored. In addition, the blood sampling room visits have the characteristics of the day-of-the-week effect and seasonality, which are due to the registration system as well as human behavior. For example, people tend to visit the blood sampling room at the beginning of the week instead of at the end. Therefore, the prediction of the specific demand of the outpatient blood sampling department is equally important for staff scheduling and resource allocation [5].

The most commonly used methods in the field of medical service prediction are linear regression models and time series analysis. Boyle et al. [10] established a general linear regression model with 11 dummy variables for predicting monthly patient admissions, which performed best. Besides, Wargon et al. [11] adopted a generalized linear model (GLM) based on calendar variables to forecast daily visits in four EDs in Paris. However, it can be very difficult to build an accurate regression model in the blood sampling room, because it is hard to ascertain which of the various influencing factors are needed in forecasting the number of visiting patients.

Time series analysis fits in mainly with the data sequence that is changing over time (e.g., the number of visiting patients). Time series models can help administrators estimate how the sequence of observations will continue into the future. They use only information on the variable itself to forecast visits trend. Time series models used for forecasting include autoregressive moving average (ARIMA) models, exponential smoothing models, and structural models. Among them, the ARIMA model, which proved to be relatively fast with a small calculation amount and have high reliability as well as accuracy, has been widely used [9, 12]. Ibrahim et al. estimated an ARIMA model that can realize the three-month forecasting of incoming patients in an OPD medical laboratory [13]. Chen et al. [14] and Kadri et al. [15] applied ARIMA to predict ED visitor volume and daily patient attendances, respectively. Compared with other models, the univariate ARIMA model achieved the best performance in predicting Hospital Medicine (HM) patient volume [16]. For the time series of the monthly incidence of hepatitis B in China with a downward trend and a strong periodicity, the ARIMA model has better fitting and predictive performance than the GM (1,1) model [17]. In predicting the number of emergency room patient arrivals per hour, ARIMA outperformed the Holt Winters, TBATS, and neural network methods [18]. In general, studies have shown that the ARIMA model, which assumes that future data values are linearly dependent on current and past data values, is suitable for predicting linear and stationary time series data.

The simple exponential smoothing (SES) model is a forecasting model that likewise estimates the historical forecast value based on the time series data. It assumes that the historical data and forecast data of the time series data are relatively continuous and have a common repeating pattern, and thus they can be matched well with short-term forecasts [19]. The model is simple to understand, reducing the work and expertise required to identify the appropriate model, which is particularly suited for busy hospital managers [20]. Apart from that, it is capable of modeling trend, autoregressive, and moving average processes. As a common time series forecasting tool, the simple exponential smoothing model is applied by Bergs et al. [21] to model monthly ED visits. It was also used to predict the number of hospitalized people in 2018 [22].

Some other time series methods were also applied in forecasting hospital visits. Au-Yeung et al. [23] predicted patient arrivals in an accident and emergency department via a structural time series model. Tricahya and Rustam [24] used Fuzzy Time Series (FTS) to forecast the amount of pneumonia patients. In some cases, the time series model may give more accurate forecasts than a regression model. In Dasyam et al.'s [25] study, the authors found that the ARIMA model is more suitable for predicting wheat production in India than the parametric regression model. Coincidentally, in Yamak et al.'s paper [26], the results showed that the ARIMA model gave better results than the deep-learning-based regression models when using Bitcoin's price data set as the time series data set and making predictions accordingly.

At the same time, many studies in recent years have focused on the use of machine learning techniques for predictive modeling. Yousefi et al. [27] stated that better forecasts are viable by using the abilities of machine learning algorithms. He proposed a deep machine learning approach to predict the number of visitors in EDs for a forecasting horizon of up to 7 days. Results indicated that the proposed approach outperformed statistical alternatives available in the literature such as multiple linear regression, ARIMA, support vector regression, generalized linear models, generalized estimating equations, seasonal ARIMA, and combined ARIMA and linear regression. Among the machine learning methods, artificial neural networks (ANNs) are the most commonly used methods. In Sukmak et al.'s study, two ANN models, namely, radial basis function (RBF) and multi-layer perceptron (MLP) networks, were constructed to predict the monthly visits of anxiety disorder patients [3]. In Lu et al.'s study, five hybrid algorithm combing models were assessed and the results showed that the GM-ANN model provided the most precise prediction in forecasting the incidence of occupational diseases in China [28]. Khaldi et al. [29] found that ANN assisted with EEMD outperformed the benchmarking models for approximation and generalization capabilities, while overcoming the problem of overfitting. With the rise of machine learning methods, research comparing them with traditional prediction methods has gradually increased. However, research has showed that ARIMA outperforms ANN on data sets with higher linearity [30], and for a trending and cyclical data pattern, a simple traditional method performs well [31].

Among so many prediction approaches, the prediction efficacy differs. Extensive research conducted by forecasters indicates that there is no universally superior forecasting method [32], and theoretical arguments can be made in favor of multiple methods given the same set of data. Marcilio et al. [33] used time-series methods including generalized linear models, generalized estimating equations, and seasonal ARIMA to forecast daily ED visits. Kim et al. [16] applied exponential smoothing, ARIMA, seasonal ARIMA, and generalized autoregressive conditional heteroscedasticity (GARCH) methods in patient volume forecasting. Navares et al. [34] conducted ensemble methods, boosting methods, artificial neural networks (ANN), and ARIMA to forecast the number of daily hospital admissions to emergency services due to circulatory and respiratory cases. In short, data sets with different characteristics match different models and methods. Wang et al. [35] pointed out that the difference operator and seasonal difference operator, bases of ARIMA and SARIMA, respectively, can be used to remove the trend and seasonal component of a time series so that the original nonstationary time series could be transformed into a wide-sense stationary time series, which could then be handled by the Box-Jenkins methodology (ARMA). From this, we can learn that the ARIMA model is especially suitable for seasonal data. Besides, it can be found that the exponential smoothing model matches well with the day-of-week effect from the previous literature. For instance, Kuroda et al.'s study [36] showed that a revised forecasting model (exponential smoothing) considering “a day-of-the-week index (DWI)” was proven to have smaller prediction errors; Ahmadi et al.'s study [37] indicated that the exponential smoothing method appeared to be best suited for data with characteristics of two seasonality factors, one for the week-of-the-semester effect and one for the day-of-the-week effect.

However, since the patient arrival pattern is influenced by human behavior, the relationship between the patient flow pattern and input features (i.e., contributing variables or covariables) contains high complexity. Therefore, no matter whether it is a traditional prediction method or a machine learning method, a single prediction model might not perform well. In order to overcome this limitation, the combined model has been proposed. The basic idea of combining forecast models is to utilize each model's unique features to capture different patterns in the data, reducing prediction errors and data set bias as well as improving overall prediction accuracy [30, 38, 39]. Hibon and Evgeniou's study [40] showed that the best combinations perform better than the best individual forecasts, and the combined forecasts reduce the risk in practice of selecting an individual forecasting method. Therefore, the literature that uses combined models to complete medical demand forecasting is increasing. For example, Klute et al. [31] conducted a hybrid prediction model with better prediction performance which applied machine learning methods (XGBoost) that are good at processing periodic data and traditional methods (linear regression) that are good at processing trending data. Combining the SARIMA model used to capture the periodicity and autocorrelation of daily time series data with the SES model used to consider the day-of-the-week effect in a weekly time series, a well-fitted hybrid model for daily outpatient volume forecasting was built [6]. Combining a univariate model that captures trends, seasonality, and nonstationarity with a multivariate model that captures causal relationships of the upstream demand effects, a hybrid model which can achieve precise prediction of weekly clinic needs was proposed [4]. Hence, a promising hybrid model combining ARIMA and SES [41] will be used to predict the demand for the blood collections.

In addition, the prediction period can be divided into medium and long-term forecasts such as annual forecast, quarterly forecast, monthly forecast, weekly forecast, and short-term forecast with daily and hourly forecast intervals. Although the medium and long-term forecasts can achieve a better fitting effect, help managers make sound downstream decisions, and give more buffer time to adapt to the cyclical schedule, they cannot provide good support for the accurate arrangement of medical resources at the operational level [12, 42]. Yet, precise short-term forecasts can provide managers with theoretical support for arranging human resources as well as adequate preparation for emergencies, which tends to be more useful [43]. However, the complexity of daily time series prediction is high because the shorter the prediction period is, the lower the prediction accuracy is [38, 43]. Abraham et al. [44] showed that forecasting patient flow in EDs beyond seven days does not yield reliable results. Similarly, Marcilio et al. [33] found that a forecast horizon 7 days ahead yields much better results than that which is 30 days ahead. Thus, we will develop a daily outpatient blood collection forecast with a 7-day forecasting horizon, with the aim of fully considering the characteristics of the daily cycle time series and improving the prediction accuracy.

Taking into account what we have discussed above, it can be concluded that model selection, prediction period, and prediction accuracy are the three key issues that need to be considered in the establishment of a prediction model. In terms of model selection, mining the key time series features of data is the premise of establishing accurate prediction models. For time series data of different features, different prediction models ought to be used [31]. The daily number of blood collections, our research object, is a seasonal nonstationary time series with trend and periodicity [5, 19]. The prediction method must accurately capture the characteristics of trend, periodicity, and randomness. The ARIMA model, as a common method for modeling time series with high prediction accuracy, not only considers long-term trends, seasonal effects, and random fluctuation effects [45] but also has the ability to process nonstationary time series data for prediction [13]. Therefore, we will first use the differential ARIMA model to predict the number of blood collections. Additionally, research shows that the prediction accuracy of the model that takes into consideration the day-of-the-week effects is higher. In other words, medical demand from historical weekdays (such as Wednesday) can provide a more accurate forecast for the same weekday in the future [8]. In this article, we assume that historical data and forecast data of time series data have relative continuity and repetitive patterns in the medium and long-term range. Therefore, a SES model [19] will be used to capture the day-of-the-week effects of the daily number of blood collection time series, analyzing the complex uncertainty of medical service demand. Finally, we will form a combined forecasting model based on the above two models.

The research structure of the paper is as follows: Section 2 briefly introduces the research objects and methods of the paper.  Section 3 details the model and prediction results. Following that, Section 4 discusses the selection of the prediction model, the prediction period, and the prediction accuracy.  Section 5 points out the limitations of the study. Finally, the study is concluded in Section 6.

## 2. Materials and Methods

### 2.1. Study Sample

Our study selected the daily outpatient blood collection data series of a large-scale general hospital in Sichuan Province as the prediction research object, including 426 consecutive blood collection work days from March 1, 2018 to April 30, 2019.

**Figure 1:**
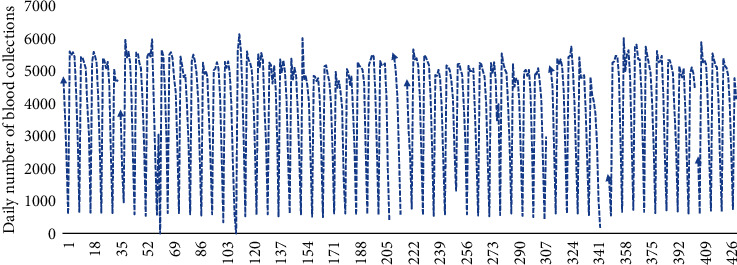
Daily time-series data of the number of blood collections in March 2018-April 2019.

**Figure 2:**
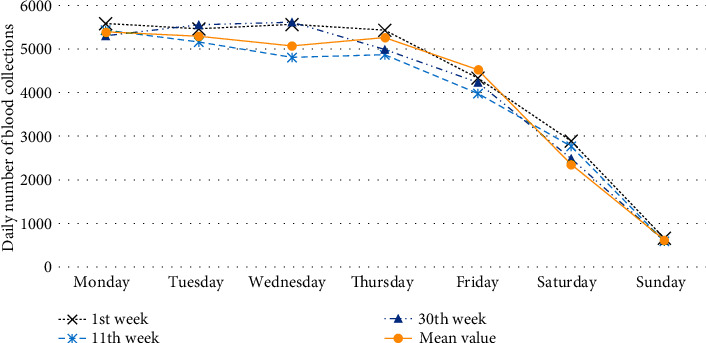
Daily number of blood collections in weekly time series.

Figure 1 shows the daily outpatient number of blood collections in the hospital with an average of 3952.6 and a standard deviation of 1702.36. The time plot shows annual seasonal fluctuations in blood sampling volume; its fluctuations have obvious holiday effects, and there exists abnormal points. Picking a few weeks at random, the numbers of the blood collections of the same day in each week are shown in Figure 2, which indicate that there are obvious trends and certain patterns during the week, that is weekly seasonality. The number of blood collections on weekdays is significantly higher than that on weekends. Besides, Figure 2 shows that daily patient volumes are typically elevated on Mondays, and the amount of blood collected on Sunday is much lower than those collected on the other six days of the week. The fluctuation in number of blood sampling room visits of every day among different weeks is small. Descriptive statistical analysis shows that the daily number of blood collections is a nonstationary time series with obvious periodic characteristics and seasonal trends.

Since the daily number of blood collections is a nonstationary time series with periodicity, a differential ARIMA model is adopted [46]. At the same time, considering the trend-of-the-week time series data, a SES model is used to capture the changing trend of the number of blood collections within a week [47]. And combined models are used to improve model accuracy and to mine more data feature information.

### 2.2. Variables and Combined Models

Considering the comprehensive characteristics of the daily number of blood collection time series, this paper constructs a weighted hybrid prediction model combining the ARIMA model and the SES model based on the idea of residual correction to carry out a daily outpatient blood collection forecast with a 7-day forecasting horizon. For every day of the week, Mondays through Sundays, we established 7-day combination forecasting model for any day of the week. The variables are defined as follows:

1. ${\overset{⌢}{Y}}_{t}^{1}$ represents the estimated value of the daily number of blood collections on day *t* which is estimated by the ARIMA model, and ${\overset{⌢}{Y}}_{t^{\prime },\tau }^{2}$ represents the predicted value of the daily number of blood collections on day *τ* at week *t*′, which is estimated by the SES model. ${\overset{⌢}{Y}}_{t}^{1}$ and ${\overset{⌢}{Y}}_{t^{\prime },\tau }^{2}$ are predictions of blood sampling room visits on the same day.
2. The combined model based on the residual correction is as follows:

(1) $$\begin{array}{c} {\overset{⌢}{Y}}_{t^{\prime },\tau }=W_{1}{\overset{⌢}{Y}}_{t}^{1}+W_{2}{\overset{⌢}{Y}}_{t^{\prime },\tau }^{2}, \end{array}$$

(2) $$\begin{array}{c} t=7*\left(t-1\right)+\tau , \end{array}$$

where ${\overset{⌢}{Y}}_{t^{\prime },\tau }$ is the weighted combined prediction value (predicted value of day *τ* at week *t*′), *W*_*i*_ is a prediction weighting coefficient of a single model *i*(*i* = 1, 2).

### 2.3. ARIMA Model

ARIMA is an extension of the autoregressive moving average model (ARMA), combining difference operation and the ARMA model. The seasonal ARIMA model is used to model a series with significant seasonal effects (periodic effects), which is transformed into a stable one through trend difference and seasonal difference, along with the fitting of the ARMA model [48].

The structure of the ARIMA (*p*, *d*, *q*) model is as follows [7]:

(3) $$\begin{array}{c} \Phi \left(B\right)\nabla^{d}{\overset{⌢}{Y}}_{t}^{1}=\Theta \left(B\right)\varepsilon_{t}, \end{array}$$

where *t* represents time; *d* is the difference order used to extract the trend information; *B* is a backward shift operator, $\left(B\right){\overset{⌢}{Y}}_{t}^{1}={\overset{⌢}{Y}}_{t-1}^{1}$; and *ε*_*t*_ is a white noise sequence, while *p*, *d*, and *q* represent the autoregressive order, difference order, and moving average order, respectively.

ARIMA modeling and prediction include three steps: model identification, parameter estimation, and model diagnosis as well as model prediction. First, the unit root test is used to determine whether the sequence is stationary. For nonstationary sequences, logarithmic transformation and difference are used to eliminate the effects of seasons and trends to obtain a stable time series. Then, the model with the smallest Bayesian information criterion (BIC) and the largest log likelihood function (log likelihood) is the optimal ARIMA model. When the model is diagnosed, the residual sequence should be white noise, and its autocorrelation coefficient (ACF) and partial autocorrelation coefficient (PACF) should not be significantly different from 0. Besides, the Ljung-Box Q statistic should have no statistical significance, and the model coefficient estimates should be statistically significant [47].

### 2.4. SES Model

The exponential smoothing method uses the weighted average of the past values of the series to predict the future value. By giving a larger weight to the recent observations and a smaller weight to the actual values in the future, the predicted values can not only reflect the latest information but also reflect the historical data information, so that the prediction results are more in line with reality [49]. Thus, an exponential smoothing method is used to model and predict the time series of the daily outpatient number of blood collections of the same day in different weeks. It can be expressed as follows:

(4) $$\begin{array}{c} {\overset{⌢}{Y}}_{t^{\prime }+1,\tau }^{2}=\alpha y_{t^{\prime },\tau }+\left(1-\alpha \right){\overset{⌢}{Y}}_{t^{\prime },\tau }^{2}, \end{array}$$

where *α* is a smoothing constant, ranging from 0 to 1, ${\overset{⌢}{Y}}_{t^{\prime },\tau }^{2}$ is the smoothed value of the daily outpatient number of blood collections of day *τ* in week *t*′, and *y*_*t*′,*τ*_ is the observed value of the daily outpatient number of blood collections of day *τ* in week *t*′. The smoothed value of the daily outpatient number of blood collections of day *τ* in week *t*′ + 1 is selected as the predicted value.

We choose the *α* value that minimizes the mean square error (MSE) and the mean absolute percentage error (MAPE) between the predicted value and the actual observed value as the smoothing coefficient, and the mean value of the observation in the first three weeks (${\overset{⌢}{Y}}_{1,\tau }^{2}$) of the same sequence is selected as the initial value of exponential smoothing, carrying out exponential smoothing prediction.

### 2.5. Prediction Error Measurement

In order to better capture the short-term autocorrelation, periodicity, and the day-of-the-week effect of the two models, we propose a combined forecasting model where a weighted average is used based on the idea of two groups of single-model residual corrections. The coefficient is determined on the basis of the comprehensive model error index. The mean absolute prediction error (MAPE) represents the average of the absolute difference between the predicted value and the observed value, which is expressed as a percentage. The advantage of using MAPE is that it facilitates the direct comparison of forecast accuracy across multiple time series. In our case study, forecast accuracy for each model was measured by calculating MAPE. The smaller the MAPE value [50], the better the model's prediction result fits:

(5) $$\begin{array}{c} MAPE=\frac{100\%}{N}\times \sum \left|\frac{Y_{t}-{\overset{⌢}{Y}}_{t^{\prime },\tau }}{Y_{t}}\right|, \end{array}$$

where *Y*_*t*_ denotes the observed blood sampling room visits on day *t*, and ${\overset{⌢}{Y}}_{t^{\prime },\tau }$ denotes the predicted blood sampling room visits at the same day.

## 3. Result

### 3.1. Data Preprocessing

Because the daily outpatient number of blood collections has a periodicity over the period of a week, the data from incomplete cycles are excluded. The time series from week 2 to week 61 are used for model building. Specifically, the data from weeks 2 to 53 were fitted with a model (from March 5, 2018 to March 3, 2019), and the data at weeks 54 to 61 were used to test the prediction results (from March 4, 2019 to April 28, 2019). This includes the time series of 52 weeks for model fitting and the time series of 8 weeks for verification while predicting each week of the last 2 months based on all observations 1 week before. We explain the process of how to predict the daily outpatient visits in the 61st week of the time series in detail, and so on, for each of the remaining 7 weeks. From Figure 1, it can be seen that there are obvious holiday heterogeneous points in the data. Thus, 13 singularities are identified from weeks 2 to 60 which are distributed outside of the scope of 2 times the standard deviation from the mean value in each day-of-the-week time series. According to the cyclical characteristics in weeks, we replace the singularity with the average value of the same point data in the previous and second periods [33].

### 3.2. Establishment of ARIMA Model

#### 3.2.1. Data Analysis and Difference Processing

**Figure 3:**
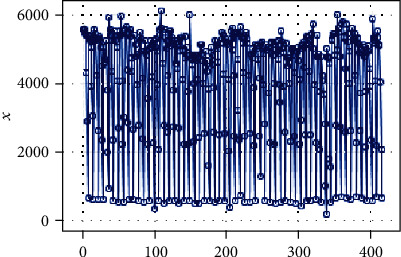
Time series data of the number of blood collections.

**Figure 4:**
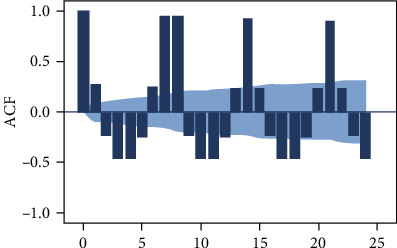
Autocorrelation coefficients of the original time series of the number of blood collections.

**Figure 5:**
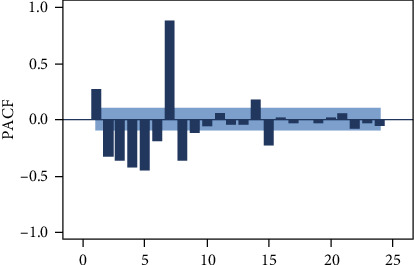
Partial autocorrelation coefficients of the original time series of the number of blood collections.

**Figure 6:**
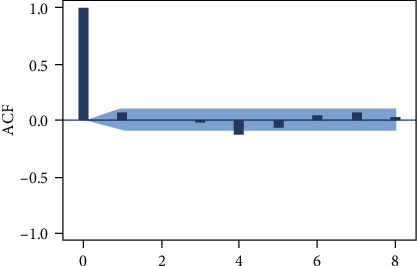
Autocorrelation coefficients of the original time series of the number of blood collections after difference.

**Figure 7:**
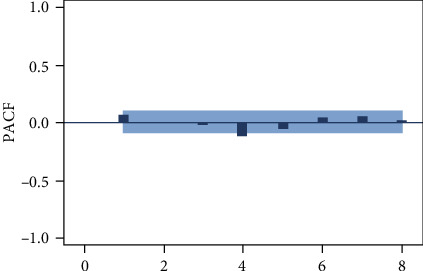
Partial autocorrelation coefficients of the original time series of the number of blood collections after difference.

According to the autocorrelation graph, which is used to determine the stationarity of the daily data series (Figures 3, 4, and 5), this series is a nonstationary series and has strong periodic characteristics with a unit of seven days. Select the first-order seven-step difference to the original sequence to extract the deterministic information in the original sequence. The difference autocorrelation and partial autocorrelation diagrams are shown in Figures 6 and 7. Then, we use the white noise test to determine the difference sequence as a nonrandom sequence and perform ARIMA model fitting and prediction on it.

#### 3.2.2. Model Identification and Parameter Estimation

According to the BIC criterion, the model with the minimum value of the BIC function is selected as the optimal model, that is, the ARIMA (0, 2, 7) model. The least squares estimation method was used to estimate the model parameters, and the parameters that did not pass the significance test were adjusted to obtain the parameter estimates (Table 1). The *p* value of the *t*-test was less than 0.05, and the model parameters constructed were statistically significant. At the same time, the model residuals are tested. The significance level *p* value of the Ljung-Box is less than 0.05. The residuals after deducting the model are not white noise sequences, and the *p* value of the LB test statistics delaying the 12th, 18th, 24th, and 30th order is significantly greater than 0.05, indicating that the model is basically reasonable and feasible (Table 2).

#### 3.2.3. Model Prediction

**Figure 8:**
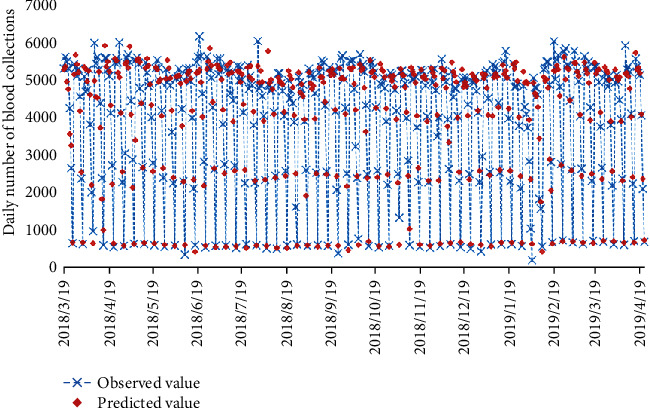
Fitted and predicted results using the ARIMA model in the blood sampling room during the 61st week.

The short-term prediction is performed on the sequence according to the fitted model, and the 7-day predicted value is obtained (see Figure 8). The average absolute percentage error (MAPE) of the fitted model is 3.32%.

### 3.3. Establishment of the SES Model

Based on the significant periodic characteristics in units of weeks presented by the time series, the exponential smoothing method is used to model the time series of different periods (weeks) at the same point in time (Sunday), and the initial value and smoothing constant *α* of the exponential smoothing are determined by referring to the method mentioned in Section 3.2. According to formula (4), we obtain the predicted value, and the average absolute percentage error of the fitting is 3.72%. The prediction result of the model is shown in Table 3.

### 3.4. Building the Combination Model

In order to make full use of the time series information and improve the prediction effect, this paper builds a combined prediction model based on the ARIMA and SES models. According to the idea of residual correction, the weighting coefficients of different weeks in the same week are calculated, respectively, and *W*_1_ = 0.73 and *W*_2_ = 0.27 are obtained. The combined prediction model is equation (6). The predicted values are presented in Table 3, while residual comparison between the ARIMA, SES, and combinatorial models are presented in Table 4. The prediction accuracy show that the combined model is better than the single model during the 61st week.

(6) $$\begin{array}{c} {\overset{⌢}{Y}}_{61,\tau }=0.73{\overset{⌢}{Y}}_{t}^{1}+0.27{\overset{⌢}{Y}}_{61,\tau }^{2}. \end{array}$$

In addition, the prediction of the remaining 7 weeks can be computed using the same approach and process, through which the prediction results are finally acquired (Table 5). As seen above, in the two empirical studies, the result of the analysis shows that the combinatorial model has superior prediction performance, with a small mean of residual errors and residual variance.

## 4. Discussion

The management of the outpatient blood collection department is one of the important indicators reflecting the level of hospital management. The prediction of the demand for blood collection can provide a reliable basis for the configuration of outpatient medical staff, which is of great significance for improving economic and social benefits. According to the needs of the hospital for the prediction system, this article takes the daily number of blood collections in a large general hospital in Sichuan Province as the data resource, and analyzes and predicts the 420 daily number of blood collections from March 2018 to April 2019 to establish a blood collection demand forecasting system. Due to the multiattribute characteristics of the daily blood collection time series data, we selected to describe a reasonable model. Besides, based on the idea of residual error correction, a combined model is designed to deeply mine the comprehensive information of the data, which can better achieve a precise prediction with a 7-day forecasting horizon. The results show that the method can improve the prediction accuracy of the number of blood collections, and the model has the ability to predict the daily blood collection demand, which is helpful for decision makers to more effectively allocate outpatient blood collection resources and save costs.

Data feature analysis is the basis for selecting a prediction model. This means that data features should correspond to model capabilities. The time series of the daily number of blood collections with seasonal fluctuations and trends fluctuates greatly and is affected by random effects such as holidays. At the same time, it shows a significant decline trend within the week. The amount of blood collection on Sunday is much lower than the other six days of the week. Our study confirms that daily demand for blood sampling services is characterized by weekly patterns, which is consistent with a mountain of previous research. For seasonal and weekly trending daily blood collection time series data, we chose ARIMA and SES to capture the linear and nonlinear characteristics. The ARIMA model belongs to a nondeterministic time series analysis method, which extracts seasonal components and nonseasonal components of the time series to predict future values with high prediction accuracy. The SES model gives weights of varying sizes according to the distance of the data. Recent data has a greater impact on the results, while long-term data has a smaller impact. It is suitable for data that has a linear trend and does not change over time. Besides, it can take into account the overall trend and the influence of regular changes, showing strong adaptability. All of the three models have good performance in forecasting accuracy. The ARIMA model has predicted the daily number of blood sampling with an overall MAPE value of 5.80%, whereas it is 5.16% for the SES model, and 4.57% for the combinatorial model. The MAPE values for combinatorial models were also significantly smaller than those from the ARIMA and SES models during weekdays and weekends. Compared to the combined ARIMA and ANN models [51], our proposed combinatorial model has MAPE ranging from 1.93% to 8.34% for day of the week and achieved lower forecast error for daily presentations in most days. For weekday-wise time series, the model was able to accurately predict day visits, with MAPE ranging from 1.93% to 5.27%, which is significantly lower than most of the other hybrid models, such as the EMD-PSO-BPANN model [52] and the SVR-FA model [53]. Moreover, the daily visit fluctuation of the weekend series is relatively complex, which cannot be effectively extracted by a single time series model. In our study, the combinatorial model performs well in modifying the extreme value of deviation in the daily blood sampling volume, especially for the time series of weekend. Research shows that the MAPE of the weekend's volume prediction reduces from 10.83% and 8.61% to 7.89%. Even though the combinatorial model performs better than the single ARIMA and SES models, the forecast precision is far from satisfactory. Specifically, the MAPE value of the three models varies from 1.93% to 5.27% during the weekdays, which is smaller than the weekend time series. Especially on Sunday, the daily number of blood collections is greatly affected by fluctuations in subjective characteristics, such as patients' medical habits and the holiday effect that lead to MAPE of up to 8.34%. Furthermore, we also noticed that the predicted values of the model from January to February were slightly abnormal, which may be due to the sudden decrease in blood collections during the Chinese New Year and the more frequent occurrence of emergencies. Hence, hospital management departments ought to pay more attention to the rational allocation and deployment of resources during these months. In general, the combinatorial model takes into account a series of complex change characteristics, such as the trend of the sequence, the change of the cycle, random interference, and the trend within the week, and it bears good prediction ability. In addition, the combinatorial model is simple and practical to implement with low computational intensiveness, while being appropriate for short-term forecast horizons.

In our study, most weighting coefficients of the combinatorial model range from 0.3 to 0.7, which indicates that the ARIMA model has a significant impact on the final predictions as the SES model. Moreover, randomness, cyclicity, and the day-of-the-week effect, which were captured by the ARIMA model and the SES model, should be taken into account. Furthermore, the average value of the weighting coefficient of ARIMA (0.6) is bigger than that of SES (0.4), indicating that the time series of blood collection are more easily affected by autocorrelation factors. Meanwhile, the result reveals that the ARIMA model shows greater utility in the combined forecasts.

According to the current status of the medical system and the requirements of hospital management in China, this paper determines a prediction model with a 7-day prediction period. The blood sampling room visits in China are more planned choices, leading to the fluctuation difference within a week that is more obvious than the monthly and annual fluctuations. Thus, it is of great practical significance to predict the daily number of blood collections in a week. Additionally, in this stable macroenvironment (medical system) and microenvironment (hospital management system), the model is also applicable to other large- and medium-sized hospitals whose daily passenger flow time series have both randomness, periodicity, and weekly pattern (the day-of-the-week effect). By establishing a blood volume prediction model, the characteristics of the time series can be captured and accurate predictions can be achieved.

## 5. Limitation

Our study suffered from a few limitations. First, the daily number of blood collections of a hospital is affected by a variety of factors. In addition to the time-series characteristics of the data itself, factors such as seasonal changes, weather changes, vacation effects, and outpatient volume should be considered. Based on this correspondence, the combination prediction model can be improved to better conform to the characteristics of the data. Additionally, this best-fit model with highly accurate forecasting takes possession of specificity, which should be considered when generalizing the finding of the study to other hospitals.

## 6. Conclusions

This paper explores the average daily number of blood collection time series data of the outpatient blood collection department in Chinese large hospitals. We select the ARIMA model and the SES model to establish a linear combination prediction model of outpatient blood collection demand based on the idea of residual correction. The thesis conducts accurate prediction research on the matching of data features and model features, which considers both prediction accuracy and demand satisfaction. It can help hospital managers to formulate periodic scheduling measures for resources based on periodic characteristics and guide the extra scheduling of resources based on the regularity caused by the day-of-the-week effect.

## Figures and Tables

**Table 1:** Parameter estimation and testing of the number of blood collections after difference during the 61st week.

Parameter	Estimation	Standard error	*t* value	Approximate Pr > |*t*|	Lag
MA1,1	-0.28043	0.0355	-7.9	<0.0001	1
MA1,2	-0.24197	0.03628	-6.67	<0.0001	2
MA1,3	-0.24873	0.0358	-6.95	<0.0001	3
MA1,4	-0.23523	0.03614	-6.51	<0.0001	4
MA1,5	-0.2765	0.03597	-7.69	<0.0001	5
MA1,6	-0.23927	0.03668	-6.52	<0.0001	6
MA1,7	0.71132	0.03579	19.87	<0.0001	7

**Table 2:** Residual autocorrelation test results during the 61st week.

Lag	*χ* ^2^	df	Pr > *χ*^2^
6		0	
12	13.37	5	0.0502
18	18.38	11	0.0732
24	27.43	17	0.0520
30	30.76	23	0.1288

**Table 3:** Forecasting performance comparison of two models during the 61st week.

Model	Mon	Tues	Wed	Thur	Fri	Sat	Sun	MAPE
Observed values	5387	5155	5095	4862	4003	2401	755	
ARIMA model	5337	5147	5091	4997	4034	2369	625	3.32%
SES model	5377	5191	5120	4954	3918	2313	627	3.72%

**Table 4:** Forecasting performance comparison of the three models during the 61st week.

Within a single week	Mon	Tues	Wed	Thur	Fri	Sat	Sun	Overall	Workday	Weekend
ARIMA model	0.93%	0.16%	0.08%	2.78%	0.77%	1.33%	17.22%	3.32%	4.65%	9.28%
SES model	0.19%	0.70%	0.49%	1.89%	2.12%	3.67%	16.95%	3.72%	5.20%	10.31%
Smoothing factor	0.099	0.200	0.006	0.100	0.003	0.027	0.199	/	/	/
Combinatorial model	0.74%	0.07%	0.07%	2.55%	0.02%	1.94%	17.15%	3.22%	4.51%	9.54%
Prediction error	40	3	4	124	1	47	129	/	/	/

**Table 5:** Forecasting performance comparison of the three models during 8 weeks.

	Mon	Tues	Wed	Thur	Fri	Sat	Sun	Overall	Workday	Weekend
ARIMA model	4.40%	2.32%	2.72%	4.90%	4.60%	7.81%	13.86%	5.80%	3.79%	10.83%
SES model	5.27%	2.81%	2.39%	4.37%	4.02%	8.16%	9.06%	5.16%	3.77%	8.61%
Combinatorial model	4.35%	1.93%	2.14%	4.19%	3.60%	7.44%	8.34%	4.57%	3.24%	7.89%

## Data Availability

The data used to support the findings of this study are obtained from a general hospital (WSH) in Sichuan Province and are available from the corresponding author upon request.
